# Hydroxyapatite-Filled Acrylonitrile–Butadiene Rubber Composites with Improved Cure Characteristics and Reduced Flammability

**DOI:** 10.3390/ma17153718

**Published:** 2024-07-27

**Authors:** Magdalena Maciejewska, Przemysław Rybiński, Anna Sowińska-Baranowska

**Affiliations:** 1 Department of Chemistry, Institute of Polymer and Dye Technology, Lodz University of Technology, 16 Stefanowskiego Street, 90-537 Lodz, Poland; 2 Institute of Chemistry, Jan Kochanowski University, 7 Uniwersytecka Street, 25-406 Kielce, Poland; przemyslaw.rybinski@ujk.edu.pl

**Keywords:** acrylonitrile–butadiene rubber, hydroxyapatite, filler, flammability, silane, ionic liquid, surfactant, vulcanization, dispersibility

## Abstract

The goal of this work was to develop acrylonitrile–butadiene (NBR) elastomer composites filled with hydroxyapatite (HAP) characterized by improved cure characteristics and resistance to burning. Silane, i.e., (3-aminopropyl)-triethoxysilane, ionic liquid, i.e., 1-decyl-3-methylimidazolium bromide and surfactant, i.e., cetyltrimethylammonium bromide, were used to improve the filler’s dispersibility in the elastomer matrix and to reduce the time and temperature of vulcanization. The effects of HAP and dispersants on the cure characteristics, crosslink density and physico–chemical properties of NBR composites were explored. The additives used, especially the ionic liquid and surfactant, effectively improved the dispersion of HAP in the NBR matrix. The amount of HAP and the dispersant used strongly affected the cure characteristics and crosslink density of NBR. The optimal vulcanization time significantly increased with HAP content and was pronouncedly reduced when ionic liquid and surfactant were applied. In addition, ionic liquid and surfactant significantly lowered the onset vulcanization temperature and improved the crosslink density and hardness of the vulcanizates while impairing their elasticity. HAP and dispersants did not significantly affect the damping properties or chemical resistance of NBR vulcanizates. Above all, application of HAP considerably enhanced the resistance of vulcanizates to thermo-oxidative aging and reduced their flammability compared with the unfilled NBR.

## 1. Introduction

Acrylonitrile–butadiene copolymer (NBR) is a special-purpose rubber whose main advantage is high resistance to chemicals such as oils, fuels, and fats. NBR products are also characterized by very good vibration-damping properties. Therefore, products made of NBR are components of tires, inner tubes, and bumpers and are used to produce various types of seals and hoses for liquid fuels and oils [1].

Industrially, NBR is used in combination with various fillers that provide an appropriate mechanical strength. In the literature, there are research works on the use of silica [2], carbon black [3], and layered minerals, e.g., montmorillonite [4] or hydrotalcite [5], as NBR fillers. Applications of waste biofillers, e.g., keratin [6] or eggshells [7], as potential fillers of this elastomer have also been tested. However, those studies often did not consider the impact of the fillers used on the flammability of NBR vulcanizates and their resistance to aging or chemicals. Moreover, silica usually resulted in an increase in the stiffness of the material and a significant reduction in elasticity, carbon black resulted in a black color for the vulcanizates, and in the case of layered minerals, it was necessary to use special methods of preparing rubber compounds, e.g., in situ polymerization or solvent intercalation, to obtain composites with an exfoliated structure that were characterized by satisfactory performance properties. Therefore, it is still justified to search for new NBR fillers that will not have a negative impact on the material’s main advantage, i.e., oil and fuel resistance, and at the same time will allow composites with improved properties, e.g., anti-aging resistance or reduced flammability, to be obtained.

Hydroxyapatite (HAP) is a naturally occurring mineral composed of calcium hydroxyphosphate with the chemical formula 3Ca_3_(PO_4_)_2_·Ca(OH)_2_. Generally, HAP is the main inorganic component of bones and teeth. It constitutes a mineral scaffold of connective tissue responsible for the mechanical strength of bones. Thus, it plays a crucial role in living organisms. HAP is commercially available either from natural sources or as synthetic powder; hence, in recent years, it has found more and more technological applications. Due to its biocompatibility, bioactivity, and resistance to degradation, HAP is commonly used in medical applications such as bone tissue engineering, implants, and drug delivery systems [8]. On the other hand, owing to its high thermal stability, non-toxicity, environmentally friendly nature, and commercial availability, HAP can be successfully used in polymers, including elastomer composites [9,10].

Regarding elastomer composites, there are some reports in the literature on the applications of HAP, mainly in natural rubber latex [11], silicone rubber [12], or carboxylated acrylonitrile–butadiene rubber [13]. Most of them concern rubber composites for biomedical applications. Regarding applications of HAP in NBR composites, Nihmath et al. [14] studied the effect of HAP nanoparticles on the thermal stability and electrical properties of vulcanizates. Incorporation of HAP increased the thermal decomposition temperature and electrical conductivity of the composites. However, the effect of HAP on other functional properties of NBR has not been investigated. Bureewong et al. [15] examined NBR composites filled with HAP obtained from fish scales. The positive influence of the HAP incorporation on the vulcanization, tensile properties, and chemical resistance of NBR was reported. Nevertheless, only the effect of HAP on the toluene resistance of NBR was investigated, not including oils and fuels, which are important for the industrial applications of this rubber. Nihmath et al. [16,17,18,19] performed a series of studies on the use of HAP as a filler for chlorinated NBR (Cl-NBR) and its blends with chlorinated ethylene–propylene–diene terpolymer (Cl-EPDM), which also confirmed the positive effect of HAP on the functional properties of chlorinated rubbers.

One of the basic factors determining the reinforcing effect and activity of the filler are the degree of its dispersion in the elastomer matrix and its interaction with the elastomer. Obtaining a uniform dispersion is often a challenge because the particles of most fillers tend to agglomerate in the elastomer matrix, and the presence of agglomerates deteriorates the performance properties of vulcanizates. In recent years, commonly used dispersants or coupling agents of fillers in elastomer composites, including NBR, have primarily been silanes [20,21,22], surfactants [5,23,24], and ionic liquids [25,26,27]. For example, Kim et al. [20] used 3-(mercaptopropyl)trimethoxy silane as a coupling agent of organophilic montmorillonite in NBR and reported strong interaction between the filler and NBR matrix since silane promoted linking of OH groups on the filler surface with nonactivated double bonds in the NBR matrix. This interaction resulted in the homogeneous dispersion of HAP in the elastomer matrix and consequently, in enhanced tensile strength and modulus of the vulcanizates. In turn, Przybyszewska et al. [23] reported the use of cationic surfactants, such as alkylphosphonium and ammonium bromides, as efficient dispersing agents of zinc oxide in NBR composites, resulting in homogeneous dispersion of nanoparticles in the elastomer matrix and improved efficiency of vulcanization. Surfactants are also commonly used to improve the dispersion of organoclays, e.g., bentonite [5] and layered double hydroxides [24]. On the other hand, Laskowska et al. [26] applied alkylimidazolium ionic liquids with bis(trifluoromethanesulfone)imide anion to improve the dispersion of fumed silica nanoparticles in the NBR. Applying ionic liquids accelerated the vulcanization and increased the crosslink density of vulcanizates. Due to the improved dispersion of silica nanoparticles, vulcanizates containing ionic liquids exhibited higher tensile strength for ionic liquid content not exceeding 5 phr.

Since polymer composites filled with HAP are used primarily in bone tissue engineering, it was difficult to find reports in the literature on the use of dispersants in this type of composite, because their biocompatibility is very important, and thus, the use of auxiliaries that could adversely affect biocompatibility is avoided. Regarding elastomers, Nihmath et al. [14,16,17,18,19] used ammonium surfactants, i.e., cetyltrimethylammonium bromide and tetrabutylammonium bromide, to prepare HAP-filled elastomer composites of NBR, Cl-NBR, and its blend with Cl-EPDM. However, in these cases, surfactants were used not only as dispersants, but primarily as phase transfer catalysts and agents for controlling particle growth, since HAP was synthesized by co-precipitation and the rubber composites were prepared using both the solution method and internal mixing. In turn, Bureewong et al. [15] used bis(triethoxysilylpropyl)tetrasulfide (TESPT) as a HAP coupling agent in NBR composites; however, some agglomerates of HAP particles were still observed in the structures of the vulcanizates.

In this work, we studied the possibility of using HAP to develop NBR composites with both improved cure characteristics, i.e., reduced time and temperature of vulcanization, and enhanced functional properties, mainly thermal stability, resistance to thermo-oxidative aging, and resistance to oils and fuels. No less important was the reduction of flammability, since NBR is commonly used for rubber products working in contact with flammable liquids. The issue of NBR’s resistance to oils and fuels, as well as its flammability, despite the basic technological applications of this rubber, has often been omitted in the studies described in the literature, and mainly research results regarding mechanical properties and conductivity are presented. Therefore, we wanted to draw attention to the properties important for NBR composites. To improve the dispersibility of HAP particles in the polar NBR matrix, dispersants with different characteristics were employed, such as silane (APTES), imidazolium ionic liquid (DmiBr) and ammonium surfactant (CTAB), which are commonly used as coupling agents for fillers (silane) or templates for synthesis of HAP particles (DmiBr, CTAB). Our intention was to select dispersants that would not have a negative impact on the chemical resistance of NBR and its flammability. To our knowledge, such composites have not yet been the subject of research described in the literature.

## 2. Materials and Methods

### 2.1. Materials

Acrylonitrile–butadiene rubber (SKN3365E type) was purchased from Konimpex Chemicals (Konin, Poland). It contained 31–35 wt.% of acrylonitrile and was characterized by a Mooney viscosity of ML1+4 (100 °C):62–68. A standard sulfur curing system was used to produce vulcanizates. It contained sulfur (Siarkopol, Tarnobrzeg, Poland) as a curing agent, 2-mercaptobenzothiazole (MBT, Sigma-Aldrich, Poznań, Poland) as a vulcanization accelerator, and zinc oxide (ZnO, SSA 10 m^2^/g, particle size < 3 μm) (Huta Będzin, Będzin, Poland) along with stearic acid (St.A.) (Sigma-Aldrich, Poznań, Poland) as activators. Synthetic hydroxyapatite (HAP), i.e., calcium phosphate hydroxide with a formula of 3Ca_3_(PO_4_)_2_·Ca(OH)_2_ and a particle size of 200 nm, purchased from Sigma-Aldrich (Poznań, Poland), was used as a filler. To improve the dispersibility of HAP particles in the elastomer matrix, and thus, their activity, the following dispersants were used: (3-aminopropyl)-triethoxysilane (APTES, Sigma-Aldrich, Poznań, Poland), 1-decyl-3-methylimidazolium bromide (DmiBr, IoLiTec, Heilbronn, Germany), and cetyltrimethylammonium bromide (CTAB, Sigma-Aldrich, Poznań, Poland).

### 2.2. Preparation of NBR Compounds and Vulcanizates Filled with Hydroxyapatite

NBR composites with the general recipes given in Table 1 were prepared using a laboratory two-roll mill (David Bridge & Co, Rochdale, UK,) equipped with rolls of the following dimensions: diameter—200 mm, length—450 mm, which worked with friction of 1.0–1.2 mm. The width of the gap between the rollers was 1.5–3.0 mm and the rotational speed of the front roll was 16 min^−1^. The average temperature of the rolls during compounding was approximately 30 °C. During the preparation of each of the NBR composites, the following procedure was used. First, the rubber was masticated for 5 min. Then, the components of the rubber compounds were introduced in the following order: HAP, St.A., ZnO, MBT, and sulfur. In the case of rubber composites with dispersants, they were added before the sulfur was introduced. As a result, sheets of rubber compounds approximately 5 mm thick were obtained, which were stored in a refrigerator at 5 °C.

To prepare the plates of vulcanizates with a thickness of approximately 1 mm, the sheets of NBR compounds were vulcanized at 160 °C using a hydraulic press with electrical heating. Vulcanization of each rubber compound was carried out using the optimal vulcanization time determined from rheometric measurements.

### 2.3. Characterization of NBR Compounds and Vulcanizates Filled with Hydroxyapatite

The cure characteristics of NBR composites were recorded at 160 °C according to the ISO 6502 [28] standard procedures, using a D-RPA 3000 rotorless rheometer (MonTech, Buchen, Germany). The following parameters of the rubber compounds were determined: minimum torque during vulcanization (S_min_), maximum torque during vulcanization (S_max_), optimal vulcanization time (t_90_), and scorch time (t_02_).

A differential scanning calorimeter (DSC) (DSC1, Mettler Toledo, Greifensee, Switzerland) was employed to determine the temperature range and enthalpy of the NBR vulcanization. Measurements were carried out according to procedures described in ISO 11357-1 [29] standard. NBR compounds with a mass of approximately 20 mg were heated from –100 °C to 250 °C in an argon atmosphere at a heating rate of 10 °C/min.

The ISO 37 [30] standard was applied to examine the tensile properties of the vulcanizates. Analysis was performed using a Zwick/Roell 1435 universal testing machine (Ulm, Germany). Five dumbbell-shaped specimens with a measuring section of 4 mm wide were studied for each vulcanizate.

The Shore A hardness was measured using Zwick/Roell 3105 tester (Ulm, Germany). Measurements for disc-shaped specimens of the vulcanizates were carried out according to the standard procedures described in ISO 868 [31]. The average of 6 determinations was taken as the measurement result for each vulcanizate.

The equilibrium swelling procedure given in the ISO 1817 [32] standard was adopted to determine the crosslink density of the NBR vulcanizates. Measurements were performed for small pieces of vulcanizates with a mass in the range of 30–40 mg, which were swollen in toluene for 48 h at room temperature. The crosslink density was calculated adopting the Flory–Rehner equation (Equation (2)) [33] for the Huggins parameter of NBR–toluene interaction given by Equation (2) [34], where *V_r_* is the volume fraction of the elastomer in swollen gel given by Equation (3), where *Q_w_* is the equilibrium swelling, *ρ_r_* is the density of the rubber (g/cm^3^), and *ρ_s_* is the density of the solvent (g/cm^3^):

(1) $$\nu_{t}=\frac{ln\left(1-V_{r}\right)+V_{r}+\chi V_{r}^{2}}{V_{0}\left(V_{r}^{\frac{1}{3}}-\frac{V_{r}}{2}\right)},$$

(2) $$\chi =0.425+0.340V_{r},$$

(3) $$V_{r}=\frac{1}{1+Q_{w}\frac{\rho_{r}}{\rho_{s}}},$$

The equilibrium swelling *Q_w_* of the vulcanizates was calculated using Equation (4), where *m_s_* is the mass of the swollen sample (mg) and *m_d_* is the mass of the dried sample after swelling (mg):

(4) $$Q_{w}=\frac{m_{s}-m_{d}}{m_{d}},$$

The resistance of NBR vulcanizates to oils and fuels was examined by swelling the vulcanized specimens in gasoline (Orlen, Płock, Poland), engine oil, and hydraulic oil (Castrol, Pangbourne, UK) for 24 h at room temperature. Then, the percentage change in the sample mass caused by the action of the solvent was calculated and expressed as the percentage of the original mass (*Q*, swelling percent), given by Equation (5) [35], where m is the mass after swelling and *m*_0_ is the initial mass of the sample before swelling.

(5) $$Q=\frac{m-m_{0}}{m_{0}}\times 100\%,$$

A DMA/SDTA861e analyzer (Mettler Toledo, Greifensee, Switzerland) working in a tension mode was employed to study the dynamic mechanical properties of the vulcanizates. During measurements of the dynamic moduli, samples of the vulcanizates were heated from −100 °C to 70 °C at a heating rate of 3 °C/min. Analysis was performed using a frequency of 1 Hz and a strain amplitude of 4 µm.

The resistance of NBR vulcanizates to thermo-oxidative aging was examined following the ISO 188 standard [36]. To perform the aging process, plates of vulcanizates were stored in a drying chamber (Binder, Tuttlingen, Germany) at 100 °C for 7 days (168 h). The aging coefficient (*A_f_*), quantifying the resistance of the elastomer to prolonged thermo-oxidation, was calculated according to Equation (6) [37], where TS is the tensile strength and EB is the elongation at the break of the vulcanizates.

(6) $$A_{f}=\frac{{\left(EB\times TS\right)}_{after\;aging}}{{\left(EB\times TS\right)}_{before\;aging}},$$

A TGA/DSC1 (Mettler Toledo) thermogravimeter was employed to investigate the thermal stability of the NBR composites. A two-step procedure was adopted to perform TG measurements. First, small pieces of vulcanizates with a mass of approximately 10 mg were heated from 25 °C to 600 °C in an argon atmosphere (gas flow 50 mL/min) at a heating rate of 20 °C/min. Next, the gas was changed into air (gas flow 50 mL/min) and heating was continued up to 800 °C at the same heating rate.

The flammability of NBR composites was examined by employing a cone calorimeter (Fire Testing Technology Ltd., East Grinstead, UK). Measurements were carried out for square specimens of vulcanizates with dimensions of 100 × 100 × 2 mm. During the analysis, samples were irradiated horizontally using a 35 kW/m^2^ heat flux density.

An LEO 1450 scanning electron microscope (Carl Zeiss AG, Oberkochen, Germany) was employed to establish the degree of dispersion of HAP particles and other ingredients in the NBR elastomer matrix. SEM images were taken of vulcanizate fractures in liquid nitrogen. Prior to the measurements, fractures of the vulcanizates were covered with a thick layer of carbon.

## 3. Results and Discussion

### 3.1. The Influence of Dispersants on the Hydroxyapatite Dispersibility in the NBR Composites

**Figure 1:**
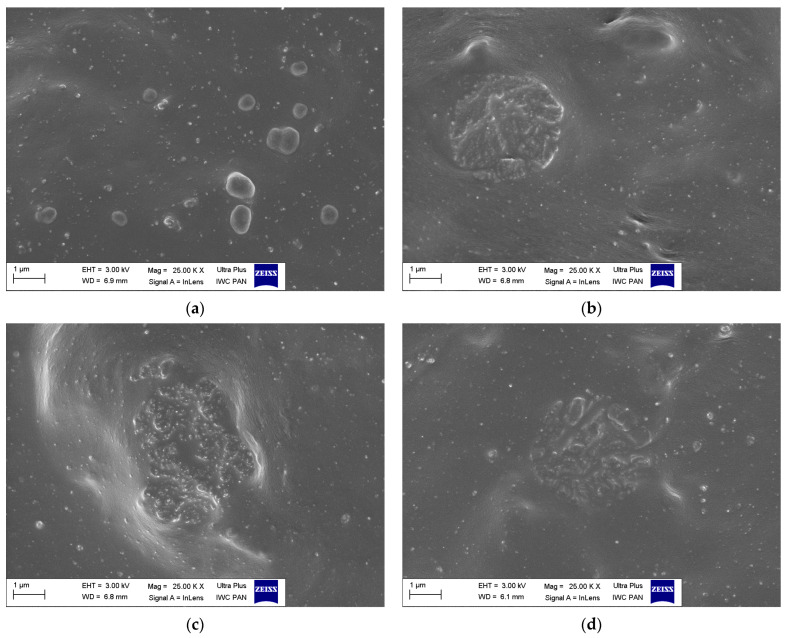

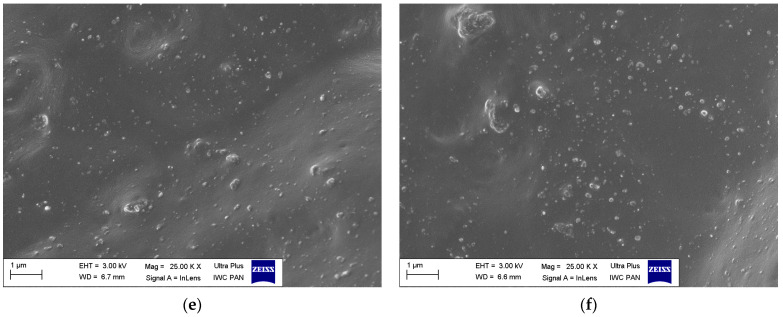
Scanning electron microscopy (SEM) images of NBR composites: (**a**) 0HAP; (**b**) 10HAP; (**c**) 30HAP; (**d**) 30HAP/APTES; (**e**) 30HAP/DmiBr; (**f**) 30HAP/CTAB.

In the first step of this research, scanning electron microscopy (SEM) was employed to examine the dispersion degree of HAP particles in the NBR elastomer matrix. Furthermore, the influence of dispersants, i.e., silane APTES, ionic liquid DmiBr, and surfactant CTAB, on the HAP dispersibility in the NBR composites was established. SEM images of vulcanizate fractures are presented in Figure 1.

Regarding the unfilled NBR vulcanizate, single agglomerates were observed heterogeneously distributed in the elastomer matrix (Figure 1a). The size of these agglomerates was approximately 1 micrometer. Since this vulcanizate contained only a crosslinking system composed of sulfur, accelerator, zinc oxide, and stearic acid, it was concluded that the agglomerates visible in the SEM image were created by the components of the crosslinking system, or a by-product of their reaction, which may have been zinc sulfide [38].

Analysis of SEM images of vulcanizates filled with HAP (Figure 1b,c) revealed the tendency of HAP particles to agglomerate in the NBR matrix. SEM images showed agglomerates with a size of 3–5 μm, and the size of the agglomerates increased with the HAP content in the composite. These agglomerates were well wetted with the elastomer and thoroughly covered with the elastomer film.

The use of APTES silane as a dispersant did not have a significant impact on the degree of dispersion of HAP particles in the NBR elastomer matrix (Figure 1d). Agglomerates were still observed in the SEM image, although their size was slightly smaller than the agglomerates present in the SEM image of the 30HAP vulcanizate, which did not contain silane. The most effective dispersant was the ionic liquid DmiBr, which ensured uniform dispersion of all components of the NBR composite (Figure 1e). It should be mentioned that imidazolium ionic liquids are often used as templates in the synthesis of HAP to control the growth and morphology of HAP particles [39,40,41]. Due to their ionic structure, ILs can interact with HAP both through ionic interactions and π–π stacking interactions. In this way, ILs can cover the HAP surface to improve the morphology and crystallinity during synthesis [39]. Similar interactions between HAP and the ionic liquid in the elastomeric matrix can prevent the agglomeration of HAP particles in the elastomer composites. The CTAB surfactant also showed satisfactory effectiveness as a dispersant, and a generally uniform dispersion of HAP was observed in the SEM image of the vulcanizate with CTAB. Single agglomerates were also present, but as their size did not exceed 1 micrometer, they were much smaller than in the case of the 30HAP vulcanizate. CTAB is one of the most popular surfactants used as a template for the synthesis of HAP particles with defined morphology. By creating micelles, it surrounds the growing particles and prevents their aggregation during the synthesis process [42,43,44]. It may also have a similar effect in an elastomer matrix.

### 3.2. The Influence of Hydroxyapatite and Dispersants on the Cure Characteristics of NBR Composites

In the next step of the study, the influence of the HAP loading on the cure characteristics of NBR composites was studied with respect to the unfilled rubber compound. Next, the effect of dispersants was discussed in relation to the NBR composite filled with 30 phr of HAP. The results of the rheometric measurements and the crosslink densities of NBR composites cured at 160 °C are presented in Table 2.

The minimum torque (S_min_) is an important parameter from a processing point of view, as it relates to the viscosity of the uncured rubber compound [45]. It is assumed that the greater the S_min_, the greater the viscosity of the uncured rubber compound, and therefore, its processing is somewhat more difficult. HAP and silane had no significant effect on the S_min_, whereas incorporation of ionic liquid DmiBr and surfactant CTAB resulted in a significant increase in the S_min_, and thus, in the viscosity of the uncured rubber compound compared with the unfilled NBR.

Applying HAP enhanced the maximum torque (S_max_) compared with the unfilled NBR. Moreover, S_max_ increased with increasing HAP content in the rubber compound. This was due to the hydrodynamic effect of the filler, which formed a rigid phase in the elastomeric matrix. The addition of dispersants increased the S_max_ value compared with 30HAP, with silane APTES having the least effect among these additives and DmiBr the greatest.

The torque increment (ΔS) values showed a similar tendency to that of the S_max_. The ΔS increased with the increase in the HAP content, which resulted from the hydrodynamic effect of the filler since HAP did not significantly affect the crosslink density (ν_t_) of the vulcanizates. Importantly, the silane, ionic liquid, and surfactant caused a significant increase in ΔS compared with the rubber compounds without these additives, due to the significantly enhanced ν_t_ of the composites containing these dispersants (Table 2). Vulcanizates with DmiBr and CTAB exhibited significantly higher crosslink density compared with the unfilled NBR and other vulcanizates filled with HAP. Thus, it was concluded that CTAB and DmiBr were able to catalyze the crosslinking reactions. On the other hand, these additives significantly improved the dispersion of the curatives in the NBR elastomer matrix, thus facilitating contact between the curatives and consequently improving the efficiency of the crosslinking reaction.

Importantly, the addition of HAP extended the scorch time (t_02_) of the composites by approximately 1 min compared with the unfilled sample, which represents a benefit for safe processing. The amount of filler and addition of silane had no significant effect on this parameter. On the other hand, ionic liquid and surfactant shortened the scorch time to 0.4 min.

The use of HAP significantly extended the optimal time of vulcanization (t_90_) compared with the unfilled NBR. The t_90_ was extended from 16 min for the unfilled NBR to approximately 28 min for the HAP-filled rubber compounds. According to Nihmath et al. [16], hydroxyl groups of HAP may promote adsorption of curatives on the HAP surface. In turn, this may reduce the activity of the curing system, particularly vulcanization accelerators, thereby reducing the rate of the vulcanization. APTES did not have a significant effect on this parameter, because the t_90_ of the rubber compound with silane was comparable to the composite without APTES. Both the ionic liquid and the surfactant significantly reduced the optimal vulcanization time, up to 4 min for CTAB and 2 min for DmiBr, respectively. Such a short t_90_ is industrially desirable and beneficial for economic reasons. This confirmed that the addition of ionic liquid and surfactant enhanced the activity of the crosslinking system, and may have resulted from both the previously mentioned improvement in the dispersion of the curatives as well as the interactions of HAP with the surfactant and ionic liquid. Both CTAB and ionic liquids have been commonly used in the HAP synthesis because they can interact with HAP particles, controlling their growth and limiting aggregation [39,42]. Thus, they can interact with HAP in the elastomeric matrix and block some of the active centers on its surface, consequently restricting the adsorption of the crosslinking system, including the vulcanization accelerator, on the HAP surface.

The beneficial influence of the ionic liquid and surfactant on the NBR vulcanization was confirmed by the DSC results, which are presented in Table 3.

Application of HAP increased the onset crosslinking temperature by 14–16 °C compared with the unfilled rubber compound, which could have been due to adsorption of the curatives on the HAP surface, as postulated in the literature. On the other hand, the amount of heat released during crosslinking, i.e., the crosslinking enthalpy, increased compared with the unfilled benchmark. The applied additives had a positive influence on the crosslinking reactions, since they significantly reduced the onset crosslinking temperature. The most pronounced reductions in the crosslinking temperature were observed for the ionic liquid and surfactant, i.e., DmiBr and CTAB, respectively. Vulcanization of the rubber compounds containing DmiBr and CTAB started at a temperature 30–35 °C lower compared with 30HAP.

Most importantly, using HAP with the ionic liquid and surfactant enabled a significant increase in the crosslinking efficiency of the NBR compounds, as evidenced by reductions in the vulcanization time and temperature, as well as an increase in the crosslink density of NBR compared with the unfilled composite and those without dispersants.

### 3.3. The Influence of Hydroxyapatite and Dispersants on the Tensile Properties and Hardness of NBR Composites

The next step of the research was to explore the influence of HAP and the applied dispersants on the mechanical properties of NBR vulcanizates. The stress at 300% relative elongation (SE_300_), tensile strength (TS), elongation at break (EB), and hardness of the vulcanizates were measured. The results are presented in Table 4.

The SE_300_ parameter correlated with the ν_t_ of the vulcanizates. Due to the lack of a significant effect on the ν_t_, HAP also had no meaningful influence on the SE_300_ compared with the unfilled vulcanizate. The TS of the vulcanizates filled with HAP was comparable to that of the unfilled NBR, since the values of TS were within the standard deviation. HAP also did not significantly affect the EB of the vulcanizates or therefore, their elasticity. Due to the hydrodynamic effect of the filler, vulcanizates filled with HAP exhibited approximately 4–6 times higher Shore A hardness compared with the unfilled benchmark. The influence of the dispersing agents on the tensile properties of the vulcanizates correlated with their effect on the ν_t_. Vulcanizate with APTES showed higher ν_t_ compared with 30HAP and thus, slightly higher SE_300_, lower EB, and higher hardness. It should be noted that applying APTES improved the TS compared with 30HAP vulcanizate. This could have resulted from both an improvement in the dispersion of the vulcanizate components, including HAP, and an increase in the crosslink density compared with 30HAP. On the other hand, due to the higher ν_t_, vulcanizates containing DmiBr or CTAB were characterized by significantly higher SE_300_ and hardness compared with 30HAP. However, increasing the ν_t_ of these vulcanizates did not result in an improvement in TS, and additionally, a significant reduction in EB (by approximately 400%) was observed compared with 30HAP, which indicated that these vulcanizates were over-crosslinked. It is commonly known that TS increases with ν_t_ to a critical value of the ν_t_. Further increase in ν_t_ causes the TS to deteriorate because the vulcanizate becomes brittle and breaks quickly [46]. Therefore, despite improving the dispersion degree of the rubber compound ingredients in the elastomer matrix, DmiBr and CTAB did not enhance the tensile properties of the vulcanizates. Hence, it would have been necessary to optimize the content of DmiBr and CTAB in the composite to obtain both an improvement in the dispersion degree of HAP and the optimal crosslink density of the vulcanizates.

### 3.4. The Influence of Hydroxyapatite and Dispersants on the Thermo-Oxidative Aging Resistance of NBR Composites

**Figure 2:**
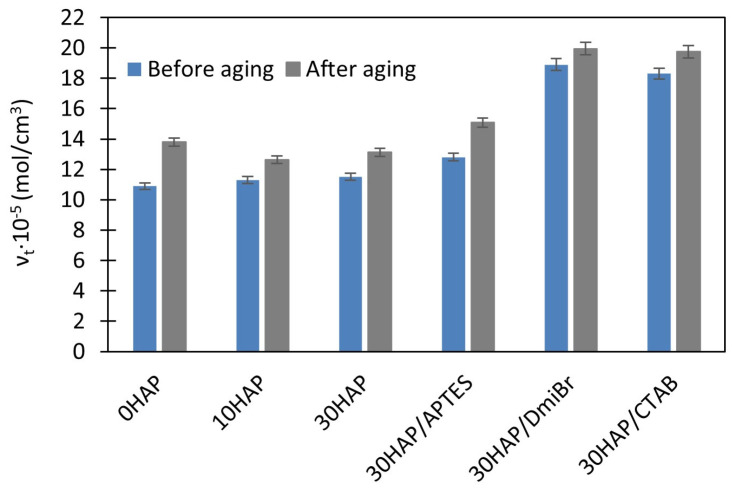
Effect of thermo-oxidative aging on the ν_t_ of NBR vulcanizates filled with HAP.

**Figure 3:**
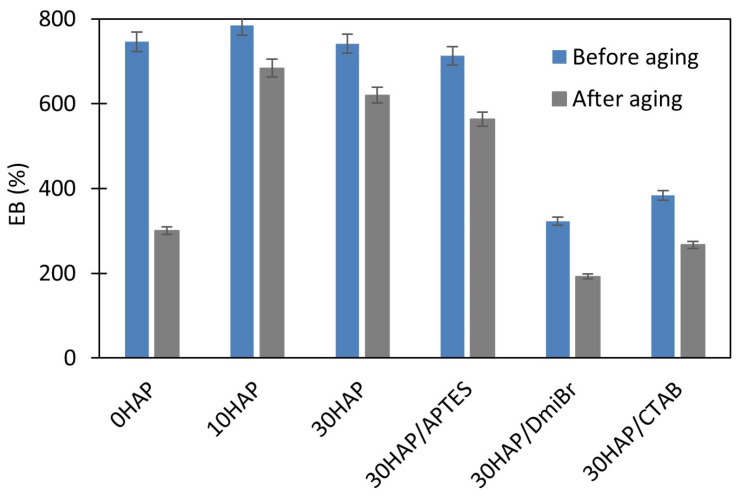
Effect of thermo-oxidative aging on the EB of NBR vulcanizates filled with HAP.

**Figure 4:**
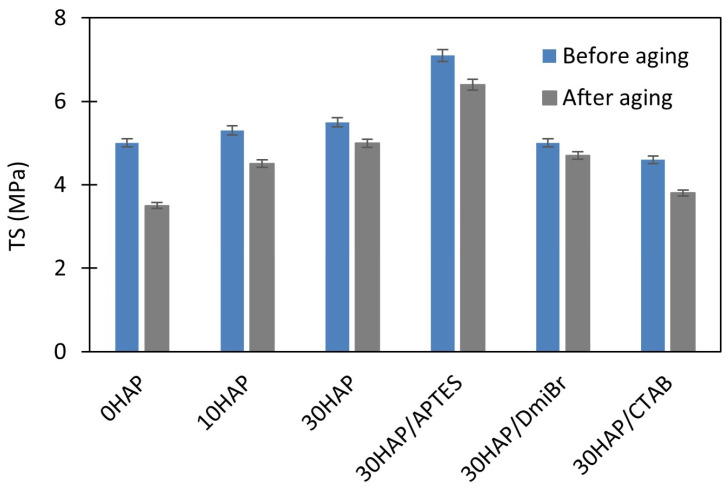
Effect of thermo-oxidative aging on the TS of NBR vulcanizates filled with HAP.

**Figure 5:**
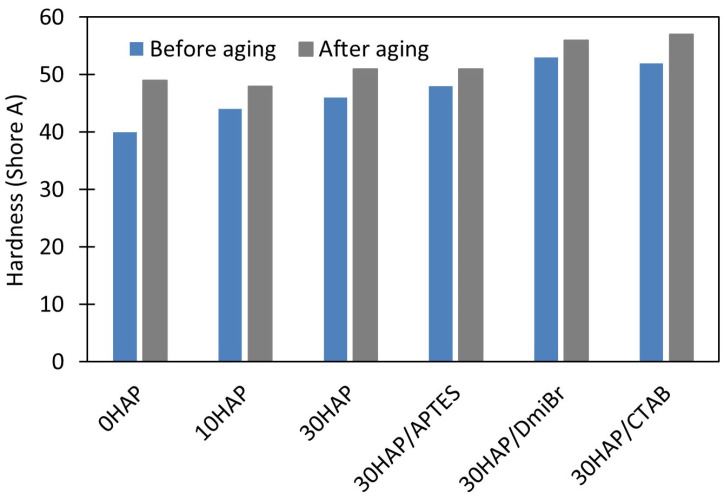
Effect of thermo-oxidative aging on the hardness of NBR vulcanizates filled with HAP.

To investigate the resistance of vulcanizates to thermo-oxidative aging, measurements of ν_t_ and mechanical properties under static conditions were used. Resistance to thermo-oxidative aging is determined by the difference in the values of these parameters before and after aging. The results are presented in Figure 2, Figure 3, Figure 4 and Figure 5.

Prolonged exposure of NBR vulcanizates to high temperature during thermo-oxidative aging (100 °C, 7 days) caused a significant increase in the ν_t_. This was particularly observed for the unfilled vulcanizate (Figure 2), and resulted from the fact that the rubber compounds were not vulcanized to the maximum crosslink density, but to the optimal ν_t_ to avoid over-crosslinking. For this reason, rubber composites usually vulcanize to a small extent during their use at high temperatures and during their thermo-oxidative aging at high temperatures. The consequences of the increase in ν_t_ of vulcanizates during aging were changes in their mechanical properties as shown in Figure 3, Figure 4 and Figure 5, i.e., a significant increase in the hardness of the vulcanizates, and reduction in their TS and EB. Most importantly, the greatest changes in mechanical properties due to aging were obtained for the unfilled vulcanizate. Hence, it can be concluded that the addition of HAP significantly improved the resistance of NBR vulcanizates to thermo-oxidative aging. This was confirmed by the aging coefficient (A_f_) values presented in Table 5. The smaller the changes in the TS and EB of vulcanizates that occurred because of aging, the greater A_f_ (closer to 1) was achieved.

The unfilled NBR was characterized by significantly lower A_f_ compared with the vulcanizates filled with HAP; thus, it exhibited poor resistance to thermo-oxidative aging. HAP highly improved the aging resistance of NBR. It should be noted that the A_f_ increased from 0.28 for the unfilled vulcanizate to approximately 0.76 for 30HAP. Hence, it was concluded that the shape of the HAP crystals and their aspect ratio, as well as their spatial distribution in the elastomer matrix, could determine the thermal behavior of the NBR vulcanizates, i.e., thermal stability, flammability, and resistance to thermo-oxidative aging. The improved thermo-oxidative aging resistance of NBR composites could be attributed to the high aspect ratio of the HAP nanostructures, which reduced the heat transfer by the crosslinked elastomer network. The homogeneously dispersed HAP particles disrupted both the oxygen supply and the heat diffusion through the sample, thus reducing the susceptibility of the material to thermo-oxidative aging [47]. In addition, the polar–polar interaction between NBR rubber chains and HAP particles promoted the creation of a strong structure, which reduced the heat diffusion and oxygen supply. It should also be noted that thermo-oxidative aging of the NBR vulcanizates increased their crosslink density, which caused deterioration of their mechanical properties, especially in the case of unfilled vulcanizate. Based on the rheometric tests, the addition of HAP caused a significant extension of the crosslinking time of NBR at 160 °C, so it can be expected that at the temperature of 100 °C at which the aging process was performed, NBR composites filled with HAP would also be much less susceptible to further crosslinking than the unfilled NBR. This shows that HAP can be a good barrier to hinder the thermal degradation, thermo-oxidative aging, and flammability of the NBR elastomer.

APTES did not affect the aging resistance, whereas vulcanizates containing DmiBr or CTAB demonstrated slightly lower A_f_ values (of approximately 0.57) compared were 30HAP, but still much better than the unfilled NBR. The weaker resistance to aging of vulcanizates with DmiBr and CTAB compared with 30HAP was because they were over-crosslinked and therefore brittle before aging. Long-term exposure to elevated temperature during the aging process resulted in further crosslinking of these highly vulcanized materials and consequently, further deterioration of their mechanical properties.

### 3.5. The Influence of Hydroxyapatite and Dispersants on the Dynamic Mechanical Properties of NBR Composites

**Figure 6:**
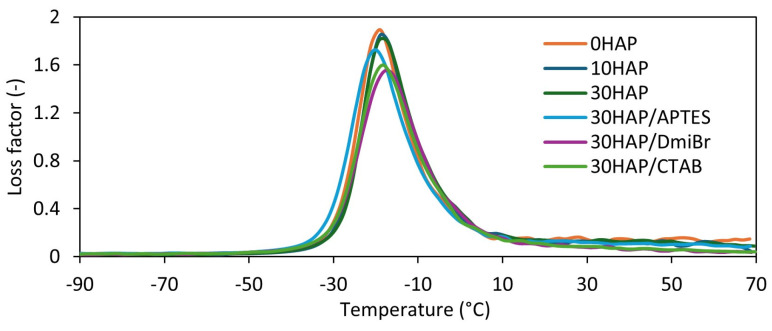
Mechanical loss factor (tan δ) as a function of temperature for NBR vulcanizates filled with HAP.

In Table 6 and Figure 6, the results of the performed DMA tests as a function of temperature are presented. Based on the DMA curves of the dependence of the mechanical loss factor (tan δ) on temperature, the glass transition temperature of NBR (T_g_) was determined. Moreover, the values of tan δ were determined at the T_g_ and in the rubbery elastic region at temperatures of 25 °C (tan δ_25°C_) and 60 °C (tan δ_60°C_), respectively.

HAP did not significantly affect the T_g_ of NBR, which ranged from −19 °C to −18 °C. Similarly, the addition of dispersants did not have a significant effect on the T_g_. Therefore, it can be concluded that neither HAP nor the dispersants used will have a significant impact on the operating temperature range of NBR vulcanizates under dynamic conditions.

Tan δ is determined as the ratio of the loss modulus to the storage modulus and represents the material’s ability to dampen vibrations. A larger tan δ indicates better damping properties of the material [48]. The tan δ at T_g_ of the unfilled vulcanizate was 1.85. HAP, regardless of its amount, had no significant effect on the values of tan δ at T_g_. However, vulcanizates containing dispersants, especially ionic liquid DmiBr and surfactant CTAB, were characterized by much lower tan δ at T_g_ compared with 30HAP. This was due to their higher crosslink density, because the greater the number of crosslinks, the more limited was the mobility of elastomer chains and consequently, the elasticity was lower and therefore, the damping properties were worse.

Regarding the dynamic properties of the vulcanizates in the rubbery elastic region, the tan δ at temperatures of 25 °C and 60 °C for the unfilled vulcanizate was 0.11. Therefore, it can be concluded that in the elastic state, the temperature did not have a significant impact on the damping properties of this material, because the differences in tan δ values were within the measurement error. HAP did not have a significant impact on the tan δ in the elastic state, nor therefore on the material’s ability to dampen vibrations. The case of silane application was similar. On the other hand, vulcanizates with DmiBr and CTAB were characterized by lower tan δ values at 25 °C and 60 °C compared with 30HAP, which may have indicated their slightly worse damping properties resulting from over-crosslinking.

### 3.6. The Influence of Hydroxyapatite and Dispersants on the Thermal Stability of NBR Composites

**Figure 7:**
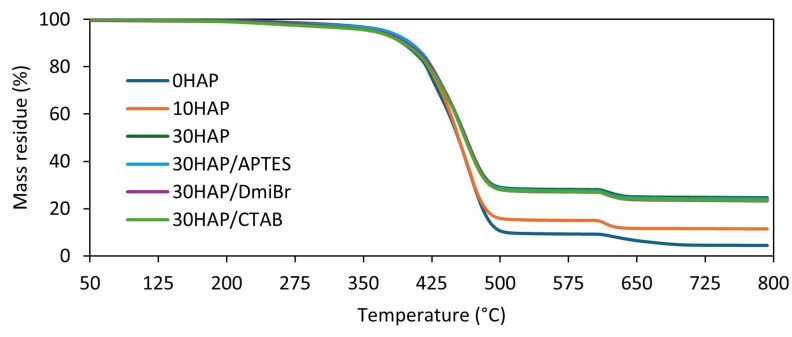
Thermogravimetric (TG) curves of NBR vulcanizates filled with HAP.

**Figure 8:**
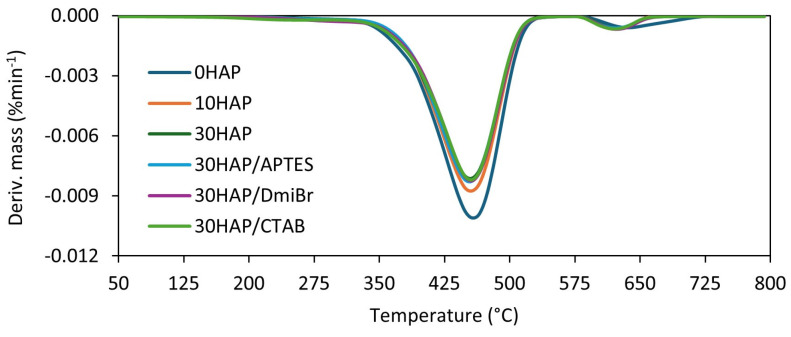
Derivative thermogravimetric (DTG) curves of NBR vulcanizates filled with HAP.

HAP is characterized by high thermal stability [49]. Therefore, in the next step of the research, the influence of HAP on the thermal decomposition of NBR composites was studied using thermogravimetry (TG). Based on the data obtained, the following were determined: the temperature at which the sample mass changed by 5% (T_5%_); the peak temperature on the curve of the mass change rate over time (T_DTG_), corresponding to the temperature at which the thermal decomposition of the material proceeded the fastest; the percentage of mass loss at a temperature of 25–600 °C (Δm_25–600°C_); the percentage of mass loss at 600–800 °C (Δm_600–800°C_); and the percentage of mass that remained at 800 °C (R_800°C_). Results are presented in Table 7 and Figure 7 and Figure 8.

HAP slightly increased the onset decomposition temperature (T_5%_) of NBR composites. A maximum increase of 8 °C was achieved for the 30HAP vulcanizate, since T_5%_ increased with the content of HAP in the elastomer composite. The addition of dispersants, i.e., DmiBr and CTAB, resulted in a reduction in T_5%_ by 10–12 °C compared with 30HAP. This was due to the significantly lower thermal stability of pure DmiBr and CTAB compared with NBR [50,51,52]. However, HAP and dispersants did not significantly affect the temperature of the maximum decomposition rate (T_DTG_), which ranged from 476 °C for the unfilled NBR to 473 °C for vulcanizates containing APTES and CTAB.

The first mass loss at temperatures from 25 °C to 600 °C corresponded to the pyrolysis of rubber and organic substances in argon. The percentage values of mass loss in this temperature range correlated very well with the HAP content. The higher the filler content, the smaller was the sample’s mass loss in this temperature range. As expected, the highest Δm_25–600°C_ was determined for the unfilled vulcanizate (approximately 91%). After supplying air to the measuring chamber of the TG analyzer in the temperature range of 600–800 °C, the residues were burned, which resulted from the pyrolysis that took place in the first stage of thermal decomposition. HAP and its content had no significant effect on the Δm_600–800°C_. After completion of the TG test, the residue at 800 °C consisted of ZnO and HAP, as well as ash remaining after burning the sample.

Most importantly, taking into account the obtained TG results, it can be concluded that HAP did not have a detrimental impact on the thermal stability of NBR vulcanizates.

### 3.7. The Influence of Hydroxyapatite and Dispersants on the Flammability of NBR Composites

Having established the influence of HAP and dispersants on the thermal behavior, we then investigated the effects of these additives on the flammability of NBR composites. Since NBR products, e.g., seals or hoses, are often used in environments with flammable liquids, their resistance to burning is very important from the point of view of fire safety. The results of the flammability tests are presented in Table 8.

It should be noted that the use of HAP reduced the flammability of NBR composites, as evidenced by lower HRR, THR, and HRC of the vulcanizates filled with HAP compared with the unfilled benchmark. Moreover, these parameters decreased with increasing HAP content in the NBR composite. The positive effect of HAP on the flame retardancy of NBR could have resulted from the reduction in the transfer of heat, gases, and gaseous products of thermal decomposition via a network formed of HAP particles dispersed in the elastomeric matrix. In addition, the polar–polar interaction between the NBR rubber chains and HAP particles may have facilitated the creation of a strong structure that reduced the flammability of the vulcanizates compared with the unfilled NBR [18]. Thus, the higher the content of HAP, the lower was the flammability of the elastomer composite, which is very important for industrial applications of NBR products. On the other hand, dispersants increased the flammability of NBR composites compared with 30HAP. These additives were organic compounds containing combustible carbon, nitrogen, and hydrogen atoms, which were the sources of the fuel and thus increased the HRR, THR, and HRC compared with 30HAP. However, it is worth noting that the flammability of composites containing dispersants was still lower than that of the unfilled vulcanizate.

### 3.8. The Influence of Hydroxyapatite and Dispersants on the Resistance of NBR Composites to Oils and Fuels

The main advantage of NBR is its resistance to oils, fuels, and fats. For this reason, the basic applications of NBR rubber are seals, washers, and hoses used in pneumatics and hydraulics [1]. Therefore, it was justified to investigate the influence of HAP and dispersants on the chemical resistance of NBR. Results are presented in Table 9.

Most importantly, HAP had no detrimental effect on the chemical resistance of NBR composites. The swelling percentages determined in engine oil, hydraulic oil, and gasoline were even slightly lower for the vulcanizates containing HAP compared with the unfilled benchmark. Moreover, dispersants did not significantly affect the oil and fuel resistance of NBR vulcanizates compared with 30HAP. Thus, HAP and the tested dispersants can be used without a negative impact on the key feature of NBR rubber that determines its industrial applications.

## 4. Conclusions

Hydroxyapatite can be successfully applied as a filler of NBR composites to strongly improve their resistance to thermo-oxidative aging, increase thermal stability, and reduce flammability, without detrimental effect on the mechanical performance, elasticity, chemical resistance, and damping properties of the vulcanizates. This is particularly important because NBR is used primarily for products, e.g., seals or hoses, operating in the environment of fuels and oils, i.e., flammable substances. HAP-filled vulcanizates were characterized by approximately 60% higher thermo-oxidative aging factor compared with the unfilled NBR, while the temperature of thermal decomposition increased by 8 °C. Regarding flammability, HAP reduced the heat release rate by approximately 33%, and the total heat released was reduced by approximately 20% compared with pristine NBR composite.

Silane (APTES), ionic liquid (DmiBr), and surfactant (CTAB) are efficient dispersants and improved the dispersibility of HAP particles and curatives in the NBR elastomer matrix. The most homogeneous dispersion of HAP achieved with DmiBr. Above all, HAP in combination with DmiBr and CTAB significantly boosted vulcanization, causing an approximately nine-fold shortening of the vulcanization time and a reduction in the vulcanization temperature by approximately 25% compared with NBR composites without these additives. This is of key importance for the industrial use of such systems to reduce energy consumption for vulcanization of rubber composites and consequently, the production costs.

As expected, HAP exhibited lower activity as a filler compared with traditional active fillers such as carbon black and silica. For this reason, vulcanizates filled with HAP showed lower tensile strength compared with those filled with silica or carbon black. On the other hand, HAP-filled NBR showed higher elasticity and consequently, much better damping properties in comparison with vulcanizates containing silica and carbon black [25,26,27,53]. In turn, NBR vulcanizates filled with HAP were characterized by better thermal stability and flammability than some vulcanizates containing silica, bentonites, or halloysite nanotubes [54,55,56]. Thus, HAP can be successfully used to enhance the thermal behavior of NBR composites.

## Figures and Tables

**Table 1:** General recipes of the NBR composites, phr (parts per hundred of rubber).

Compound	0HAP	10HAP	30HAP	30HAP/APTES	30HAP/DmiBr	30HAP/CTAB
NBR	100	100	100	100	100	100
ZnO	5	5	5	5	5	5
St.A.	1	1	1	-	-	-
Sulfur	2	2	2	2	2	2
MBT	2	2	2	2	2	2
HAP	-	10	30	30	30	30
APTES	-	-	-	3	-	-
DmiBr	-	-	-	-	3	-
CTAB	-	-	-	-	-	3

**Table 2:** Cure characteristics at 160 °C and crosslink densities of NBR composites (S_min_—minimum torque, ΔS—torque increase during vulcanization, t_02_—scorch time, t_90_—optimal vulcanization time, ν_t_—crosslink density determined in toluene).

NBR Compound	S_min_ (dNm)	S_max_ (dNm)	∆S (dNm)	t_02_ (min)	t_90_ (min)	ν_t_ × 10^−5^ (mol/cm^3^)
0HAP	0.6 ± 0.2	7.9 ± 0.2	7.3 ± 0.2	0.9 ± 0.2	16 ± 1	10.9 ± 0.2
10HAP	0.7 ± 0.1	9.1 ± 0.1	8.4 ± 0.1	1.8 ± 0.2	28 ± 1	11.3 ± 0.3
30HAP	0.8 ± 0.2	11.6 ± 0.2	10.8 ± 0.2	1.9 ± 0.2	28 ± 1	11.5 ± 0.2
30HAP/APTES	0.8 ± 0.2	15.0 ± 0.2	14.2 ± 0.2	1.5 ± 0.2	27 ± 1	12.8 ± 0.4
30HAP/DmiBr	1.5 ± 0.1	18.1 ± 0.1	16.6 ± 0.1	0.4 ± 0.2	3 ± 1	18.9 ± 0.3
30HAP/CTAB	1.3 ± 0.2	17.5 ± 0.2	16.2 ± 0.2	0.4 ± 0.2	4 ± 1	18.3 ± 0.4

**Table 3:** Temperature (T_cross_) and enthalpy (ΔH_cross_) of NBR compounds’ crosslinking determined by DSC (standard deviation: T_cross_ ± 2 °C; ΔH_cross_ ± 1.0 J/g).

NBR Compound	T_cross_ (°C)	−∆H_cross_ (J/g)
0HAP	153–243	9.7
10HAP	167–245	14.5
30HAP	169–249	13.9
30HAP/APTES	140–176	14.6
30HAP/DmiBr	129–175	15.0
30HAP/CTAB	124–191	16.6

**Table 4:** Tensile properties and hardness of NBR vulcanizates (SE_300_—stress at a relative elongation of 300%; TS—tensile strength; EB—elongation at break; H—hardness).

NBR Vulcanizate	SE_300_ (MPa)	TS (MPa)	EB (%)	H (Shore A)
0HAP	1.9 ± 0.1	5.0 ± 0.4	746 ± 38	40 ± 1
10HAP	2.0 ± 0.1	5.3 ± 0.3	785 ± 52	44 ± 1
30HAP	2.1 ± 0.1	5.5 ± 0.6	742 ± 67	46 ± 1
30HAP/APTES	2.6 ± 0.1	7.1 ± 0.6	713 ± 36	48 ± 1
30HAP/DmiBr	4.0 ± 0.3	5.0 ± 0.8	323 ± 53	53 ± 1
30HAP/CTAB	3.6 ± 0.1	4.6 ± 0.3	384 ± 27	52 ± 1

**Table 5:** Thermo-oxidative aging coefficient (A_f_) of NBR vulcanizates.

NBR Vulcanizate	A_f_ (-)
0HAP	0.28 ± 0.05
10HAP	0.74 ± 0.03
30HAP	0.76 ± 0.05
30HAP/APTES	0.71 ± 0.07
30HAP/DmiBr	0.56 ± 0.05
30HAP/CTAB	0.57 ± 0.03

**Table 6:** Glass transition temperature (T_g_) and mechanical loss factor (tan δ) of NBR vulcanizates.

NBR Vulcanizate	T_g_ (°C)	tan δ_Tg_ (-)	tan δ_25°C_ (-)	tan δ_50°C_ (-)
0HAP	−18 ± 1	1.85 ± 0.05	0.11 ± 0.01	0.11 ± 0.01
10HAP	−17 ± 1	1.83 ± 0.06	0.13 ± 0.02	0.13 ± 0.02
30HAP	−17 ± 1	1.81 ± 0.06	0.12 ± 0.01	0.12 ± 0.01
30HAP/APTES	−19 ± 1	1.73 ± 0.06	0.13 ± 0.01	0.11 ± 0.01
30HAP/DmiBr	−17 ± 1	1.55 ± 0.08	0.10 ± 0.01	0.08 ± 0.01
30HAP/CTAB	−17 ± 1	1.60 ± 0.05	0.09 ± 0.02	0.08 ± 0.02

**Table 7:** Thermal stability of NBR vulcanizates (T_5%_—onset decomposition temperature, T_DTG_—DTG peak temperature, ∆m—total mass loss during thermal decomposition, R_800°C_—the percentage of mass that remained at 800 °C).

NBR Vulcanizate	T_5%_ (°C)	T_DTG_ (°C)	∆m_25–600°C_ (%)	∆m_600–800°C_ (%)	R_800°C _ (%)
0HAP	365 ± 1	476 ± 1	90.6 ± 0.9	4.8 ± 0.5	4.6 ± 0.5
10HAP	370 ± 1	475 ± 1	84.5 ± 0.6	3.5 ± 0.6	12.0 ± 0.6
30HAP	373 ± 1	474 ± 1	71.5 ± 0.8	3.4 ± 0.4	25.1 ± 0.4
30HAP/APTES	375 ± 1	473 ± 1	72.3 ± 0.9	3.6 ± 0.4	24.1 ± 0.4
30HAP/DmiBr	363 ± 1	473 ± 1	72.6 ± 0.5	3.7 ± 0.5	23.7 ± 0.5
30HAP/CTAB	361 ± 1	473 ± 1	72.8 ± 0.5	3.7 ± 0.5	23.5 ± 0.5

**Table 8:** Flammability results for NBR vulcanizates (HRR—heat release rate; THRR—temperature of the maximum heat release rate; THR—total heat released; HRC—heat capacity).

NBR Vulcanizate	HRR (W/g)	THRR (°C)	THR (kJ/g)	HRC (J/gK)
0HAP	411	463	28.4	364
10HAP	309	467	26.6	293
30HAP	276	472	24.2	277
30HAP/APTES	340	460	25.4	330
30HAP/DmiBr	309	476	26.2	300
30HAP/CTAB	345	461	25.4	339

**Table 9:** Chemical resistance of NBR vulcanizates (Q_EO_—percentage of swelling in engine oil, Q_HO_—percentage of swelling in hydraulic oil, Q_G_—percentage of swelling in gasoline).

NBR Vulcanizate	Q_EO_ (%)	Q_HO_ (%)	Q_G_ (%)
0HAP	1.0	1.2	12.0
10HAP	0.9	1.0	11.1
30HAP	0.7	0.9	10.4
30HAP/APTES	0.8	1.0	11.0
30HAP/DmiBr	0.6	0.7	10.0
30HAP/CTAB	0.5	0.7	9.9

## Data Availability

The data presented in this study are available on request from the corresponding author due to privacy.
